# Multilayer Films Electrodes Consisted of Cashew Gum and Polyaniline Assembled by the Layer-by-Layer Technique: Electrochemical Characterization and Its Use for Dopamine Determination

**DOI:** 10.1155/2012/923208

**Published:** 2012-03-15

**Authors:** Sergio Bitencourt Araújo Barros, Cleide Maria da Silva Leite, Ana Cristina Facundo de Brito, José Ribeiro Dos Santos Júnior, Valtencir Zucolotto, Carla Eiras

**Affiliations:** ^1^Departamento de Química, Centro de Ciências da Natureza (CCN), Universidade Federal do Piauí (UFPI), 64049550 Teresina, PI, Brazil; ^2^DB, Universidade Federal do Piauí (UFPI), Campus Senador Helvídio Nunes de Barros (CSHNB), 64600000 Picos, PI, Brazil; ^3^SEDIS, Universidade Federal do Rio Grande do Norte (UFRN), Caixa Postal 1524, Campus Universitário Lagoa Nova, 59072970 Natal, RN, Brazil; ^4^Nanomedicine and Nanotoxicology Laboratory, IFSC, University of São Paulo, São Carlos, SP, Brazil; ^5^Núcleo de Pesquisa em Biodiversidade e Biotecnologia, BIOTEC, Campus Ministro Reis Velloso, CMRV, Universidade Federal do Piauí, UFPI, 64202020 Parnaíba, PI, Brazil

## Abstract

We take advantage of polyelectrolyte feature exhibited by natural cashew gum (*Anacardium occidentale* L.) (CG), found in northeast Brazil, to employ it in the formation of electroactive nanocomposites prepared by layer-by-layer (LbL) technique. We used polyaniline unmodified (PANI) or modified with phosphonic acid (PA), PANI-PA as cationic polyelectrolyte. On the other hand, the CG or polyvinyl sulfonic (PVS) acids were used as anionic polyelectrolytes. The films were prepared with PANI or PANI-PA intercalated with CG or with PVS alternately resulting in four films with different sequences: PANI/CG PANI-PA/CG, PANI/PVS and PANI-PA/PVS, respectively. Analysis by cyclic voltammetry (CV) of the films showed that the presence of gum increases the stability of the films in acidic medium. The performance of the modified electrode of PANI-PA/CG was evaluated in electro analytical determination of dopamine (DA). The tests showed great sensitivity of the film for this analyte that was detected at 10^−5^ mol L^−1^.

## 1. Introduction

The physicochemical properties of nanoscale composite are a result of molecular interaction between materials of interest, such as a conducting polymer, promoting greater structural control of the formed films [1–3]. Two of the most used methods to obtain nanostructured materials in the solid state are the Langmuir-Blodgett (LB) technique [4] and the process of layer-by-layer (LbL) assembly [5]. This last one emerges as a method of deposition of alternating layers formed through the electrostatic interaction between oppositely charged solutions, where the formation of the first monolayer on the substrate surface occurs initially through a process of adsorption. In addition to the possibility of controlling the structure formed at the supramolecular level, the LbL technique has the advantage of not requiring any sophisticated equipment or procedures such as the LB technique.

 The technological interest of several research groups in composites, and more recently in nanocomposites, comes mainly from the mechanical properties and biodegradability, which are both characteristic of natural polymers, allied to the conductive properties of some synthetic polymers, which provides a great versatility of applications in areas such as engineering, biotechnology, and medicine [6–8].

 Natural gums are macromolecules formed from units of sugars, monosaccharides, linked by glycosidic bonds resulting in natural polymers with long chains and high molecular weight [9]. The gums may originate from plants exudates (e.g., Arabic gum and cashew gum), seaweed extract (e.g., agar), animal (e.g., chitosan), seeds (e.g., guar gum), and others [7]. The gum from exudates is produced in epithelial cells confined in the ducts of the trees that are released spontaneously or induced as a defense mechanism of the plant [10, 11]. The natural gums interact with water in two different ways: by retention of water molecules (thickness effect) or by building networks that enhance the connection areas (effect of gelation). Because of these behaviors, the gums are also known as hydrocolloids [12, 13].

**Scheme 1:**
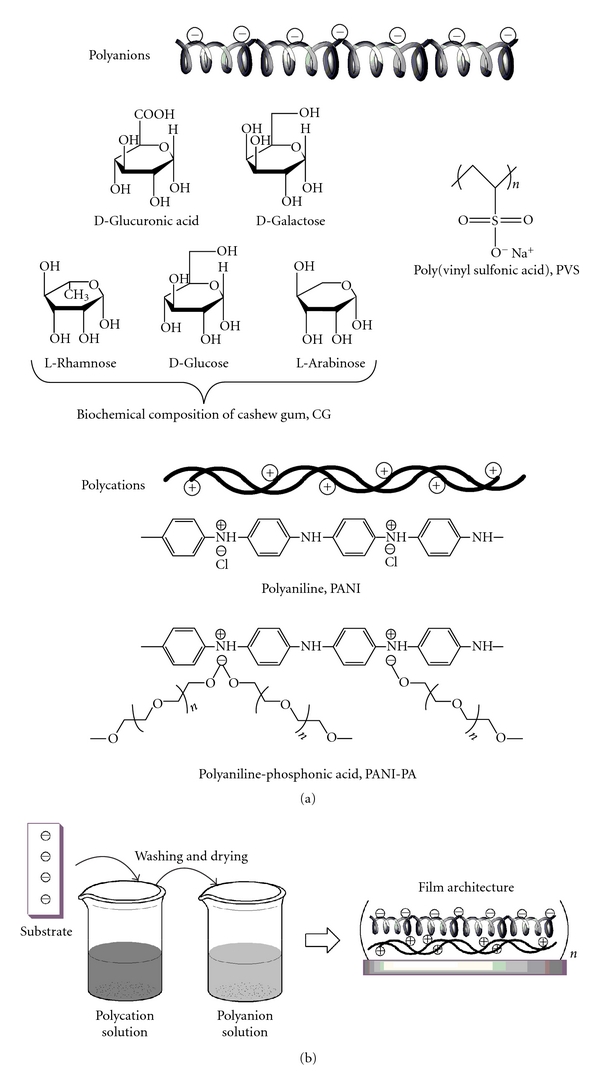
(a) Chemical structures of material employed in the films and (b) schematic process of multilayer film formation.

The cashew gum (*Anacardium occidentale* L.) is particularly interesting because it is an exudate obtained from the cashew tree, very abundant in the northeast of Brazil. It belongs to the same family of Arabic gum, widely used in the food industry, and presents similarities in their composition and in their physicochemical properties [12–14]. Cashew gum (CG) is an acidic polysaccharide complex composed of a main chain of *β*-galactose (1→3) with branches of *β*-galactose (1→6), with the terminal residues glucuronic acid, arabinose, rhamnose, 4-O-methylglucuronic acid, xylose, glucose, and mannose [14]. Brazilian gum main constituents are galactose (73%), arabinose (5%), rhamnose (4%), glucose (11%), glucuronic acid (6.3%), and residues of other sugars (less than 2%) [12, 14] (Scheme 1(a)). Additionally, the terminal glucuronic acids in the structure of the gum are responsible for the anionic nature of the material when in aqueous solution. Cashew gum has antimicrobial properties for therapeutic treatment, as well as thickening and emulsifying properties [15, 16] used in foods and drugs industry. These characteristics are due to its heterogeneous structure and high molecular weight of the polysaccharide chain, which interacts strongly with water, creating an effect of thickening or gelling in solution [16].

The polyaniline (PANI) belongs to the class of conducting polymers with high technological interest due to their potential applications as electroluminescent devices, corrosion protection, sensors, and biosensors [17, 18]. The versatility of PANI is due to changes of its oxidation state, hence its electrical conductivity, which occurs rapidly and reversibly, and its chemical stability [19, 20]. The applications of polyaniline are limited by its poor solubility in aqueous media in its conductive form [17, 20]. A proposed solution for this limitation was given by Geng et al. [21] who prepared a water-soluble conductive polyaniline through the introduction of hydrophilic dopant such as phosphonic acid (PA) in the polymeric chain. The PA is a mixture of acids mono- and bihydroxyl phosphonate at 1 : 1 molar ratio (1). The PA organic properties derive from poly(ethylene glycol) monomethyl ether. Thus, the longer the hydrophilic chain of the conducting polymer, the higher its solubility in water [21], generating new applications: 


(1)
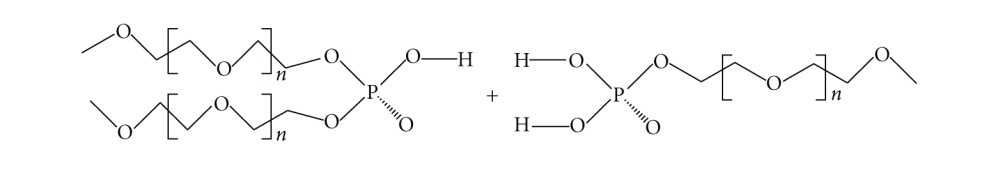



In this study, LbL films were produced with PANI or PANI-PA and the natural cashew gum (CG) in a bilayer fashion (PANI/CG)*_n_* or (PANI-PA/CG)*_n_* (where *n* is the number of bilayers). Films containing a conventional anionic polyelectrolyte, for example, poly(vinylsulfonic acid) PVS, were compared to cashew gum in the (PANI/PVS)*_n_* or (PANI-PA/PVS)*_n_* films. The films were studied through electrochemical experiments by cyclic voltammetry. We also investigated the ability of these nanocomposites to act as modified electrodes for dopamine sensing.

## 2. Experimental

Cashew gum, collected in the state of Ceará (northeast region of Brazil), was isolated and purified using sodium salt, as described by Costa et al. [22]. Afterwards 0.25 mL of ethanol was added to 5.0 g of cashew gum, which was immediately dissolved in 100 mL of Milli-Q water under stirring for 12 h, followed by filtration.

PANI was synthesized by the oxidative polymerization of aniline doubly distilled in 1.0 mol L^−1^ HCl solution containing a proper amount of ammonium persulfate ((NH_4_)_2_S_2_O_8_, Vetec). The solution temperature was kept between 0°C and 5°C, with continuous mechanical stirring. The product was maintained in ammonia hydroxide (Vetec) for 12 hours to obtain PANI in the form of emeraldine base (EB) [23]. The mixture of mono- and bihydroxyl acids designated as PA, with molecular average weight of 896 g mol^−1^, was prepared as reported by Geng et al. [21].

For the processing of polyaniline solutions, 0.47 g PANI-EB powder (with or without PA dopant) was dissolved in 25 mL dimethylacetamide (DMAc, Vetec) under stirring for 12 h. The solutions were filtered and slowly added to 26 mL of HCl solution, and the pH was adjusted at 2.8. Poly(vinyl sulfonic acid) (PVS) was purchased from Aldrich Co. and used without previous purification in aqueous solutions at a concentration of 0.5 mg mL^−1^ and pH 2.8. Ultrapure water with a resistivity of 18.3 MΩ cm (Milli-Q, Millipore) was used for preparation of all solutions. The chemical structures of the materials employed are depicted in Scheme 1(a).

Nanostructured layered films were assembled in a bilayer fashion using PANI or PANI-PA as polycationic solutions in conjunction with CG or PVS as polyanionic solutions. The deposition of each layer consisted in the immersion of the substrate in the dipping solution for 5 min, followed by rinsing in the washing solution (HCl, pH 2.8) and drying in N_2_ flow. LbL films with four distinct architectures were investigated: (PANI/PVS)*_n_*, (PANI-PA/PVS)*_n_*, (PANI/CG)*_n_*, and (PANI-PA/CG)*_n_* where *n* is the number of bilayers. Multilayer films with *n* = 2, 4,6, and 8 were obtained onto glass covered with indium tin oxide (ITO), (Scheme 1(b)).

Electrochemical measurements were carried out using a potentiostat Autolab PGSTAT30 and a three-electrode electrochemical cell with 10 mL. A 1.0 cm^2^ platinum foil and saturated calomel electrode (SCE) were used as auxiliary and reference electrodes, respectively. The LbL films onto ITO (0.4 cm^−2^) were used as the working electrode. All the experiments were performed in inert N_2_ atmosphere at 22°C in an electrolytic solution of 0.1 mol L^−1^ HCl. PANI-AP/GC LbL film containing 6 bilayers (*n* = 6) was subjected to dopamine (DA) detection using cyclic voltammetry in electrolytic solution containing 10 to 230 *μ*mol L^−1^ of DA and sweep rate of 50 m Vs^−1^. After the measurement the film tested was exhaustively washed with electrolytic solution and the reproducibility was investigated. Furthermore, the effects of the interfering ascorbic acid (AA) in the presence of DA were also studied using different proportions of AA and DA.

## 3. Results and Discussion

### 3.1. Electrochemical Characteristics of Nanostructured Films

**Figure 1:**
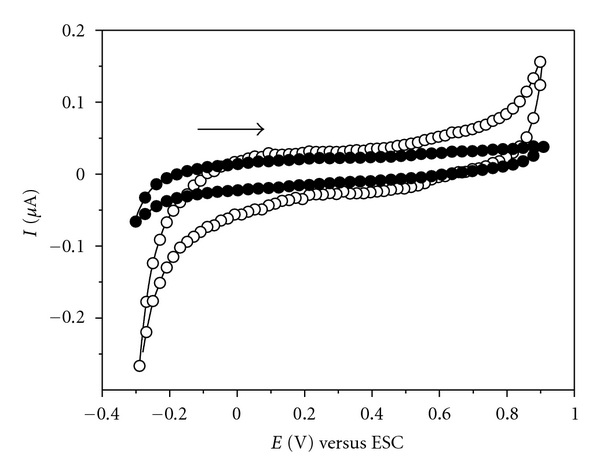
Cyclic voltammograms for bare ITO (—*⬤*—) and ITO modified with (PAH/CG)_6_ LbL films (—◯—) in HCl 0.1 mol/L at 50 mV s^−1^.

Cyclic voltammograms of ITO unmodified and modified electrodes with LbL film produced with poly(allylaminehydrochloride), PAH, and the natural cashew gum, (PAH/CG)_6_, were obtained in HCl 0.1 mol L^−1^ and are shown in Figure 1. Under our experimental conditions, it is observed that the ITO substrate has no electrochemical response to the potential range studied. However, the modified substrate with (PAH/CG)_6_ shows that the presence of the LbL film activates the electrode surface increasing the double electrical layer of this system and therefore catalyzes the processes of oxygen evolution observed in the forward sweep and hydrogen evolution observed in the reverse sweep. The increase in current values is observed in the potential in which these processes occur.

All films studied were prepared with six bilayers. A previous study about the influence of the size and nature of this anion in the supporting electrolyte for the systems studied here was carried out in HCl or H_2_SO_4_, both at 0.1 mol L^−1^, with scan rate of 50 mV s^−1^. The processes of oxidation and reduction, characteristic of conducting polymer, were shifted to more positive potentials in H_2_SO_4_ media when compared to the profile obtained in HCl solution (data not shown). The potential difference observed for this redox process was 0.05 V. According to Matveeva et al. [24], both the processes of oxidation and reduction (**E**_RED_ and **E**_OX_) and the distance between the potential at which these transitions occur are dependent on the substrate used and the size and nature of the anion of the supporting electrolyte employed. The potential shift observed for the PANI in H_2_SO_4_ reflects a limitation in the processes of charge transfer across the interface between ITO and polymeric film and also in the interface polymeric film and the electrolyte. Probably this limitation of the charge transfer process could be related to differences in mobility and the steric hindrance originated from the anionic species present in both electrolytes studied, having HSO_4_^−^ and SO_4_^2−^ for sulfuric acid and Cl^−^ for hydrochloric acid, respectively. It was also noted that the degradation processes of PANI were more intense and were best defined in H_2_SO_4_ (data not shown). This observation is likely to be explained by the fact that H_2_SO_4_ is a more oxidant acid than HCl intensifying the processes observed. Thus, the whole study presented in this paper was performed in optimized conditions of HCl media.

**Figure 2:**
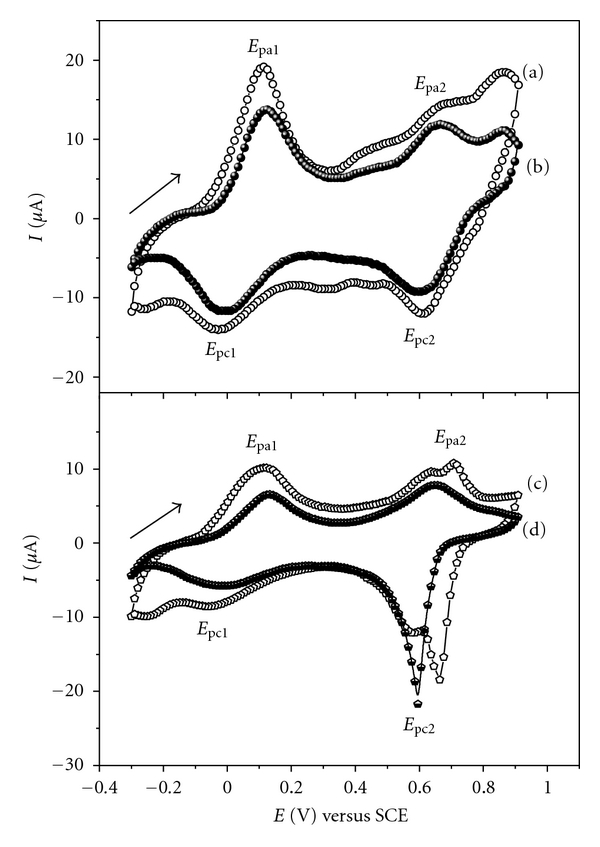
Cyclic voltammograms for LbL films: (a) (PANI-PA/PVS)_6_, (b) (PANI/PVS)_6_, (c) (PANI-PA/CG)_6_, and (d) (PANI/CG)_6_ in 0.1 mol L^−1^ HCl solution at 50 mV s^−1^.

Figure 2 shows the cyclic voltammograms obtained for the bilayers films containing the conductive polymer interspersed with cashew gum or PVS in HCl media. The electrochemical profile recorded for the LbL systems (PANI/PVS)_6_ (PANI-PA/PVS)_6_, (PANI-GC)_6_, and (PANI-PA/CG)_6_ (Figure 2) indicates the presence of two redox processes characteristic of PANI that correspond to interconversions in their states of oxidation [25]. During the direct scan there was the transition from leucoemeraldine state to emeraldine PANI, *E*_pa1_, with the expulsion of the proton, and the transition from emeraldine to pernigraniline, *E*_pa2_, was accompanied by the capture of the anion, Cl^−^. During the inverse sweep two reduction processes were observed; pernigraniline to emeraldine, *E*_pc2_, accompanied by the expulsion of the anion and emeraldine to leucoemeraldine, *E*_pc1_, which was accompanied by proton uptake.

 An intermediate process between the transitions related above is defined as acidic degradation of PANI with the formation of benzoquinone (oxidation) and hydroquinone (reduction) pair. The electrochemical behavior for the systems in Figure 2 shows that the interactions between the PANI or PANI-PA with PVS and CG do not suppress the electroactive and electrochemical properties observed and described for polyaniline. The films of PANI and PANI-PA interspersed with PVS showed the three well-known oxidation processes of PANI and a fourth oxidation process observed in the region of 0.86 V (Figures 2(a) and 2(b)). This fourth oxidation was not observed for films in which PVS was replaced by CG in the multilayer structure (Figures 2(c) and 2(d)). The oxidation process at 0.86 V can be explained as the result of an interaction between the cationic groups of PANI with anionic groups of PVS.

The phosphonic acid (PA) used in this study acts as both a modifying agent PANI, increasing its solubility, and a dopant acid promoting a further increase in current values observed in the redox processes of PANI-PA (Figures 2(a) and 2(c)). This increase in current values contributes to providing an enhancement in selectivity of the PANI-PA/PVS and PANI-PA/CG systems compared to PANI/PVS and PANI/CG, and thus the presence of PA becomes an important factor to be considered in analytical determinations using electrochemical techniques.

**Figure 3:**
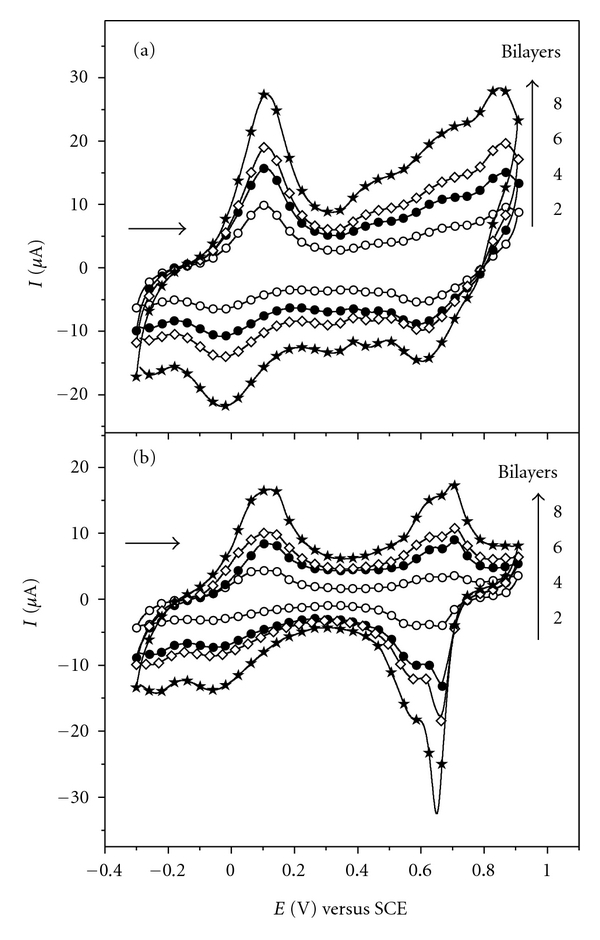
Cyclic voltammograms for LbL films from (a) (PANI-PA/PVS)*_n_* and (b) (PANI-PA/CG)*_n_*, where *n* = 2, 4,6, and 8 bilayers in 0.1 mol L^−1^ HCl solution at 50 mV s^−1^.

For the films PANI-PA/PVS (Figure 3(a)) and PANI-PA/CG (Figure 3(b)) with 2, 4, 6, and 8 bilayers, the cyclic voltammograms obtained at 50 mV s^−1^ reveal that the increase in the number of bilayers is reflected in the increase of current values. This result indicates an increment in the amount of material adsorbed on the substrate as the number of bilayers increases.

**Scheme 2:**
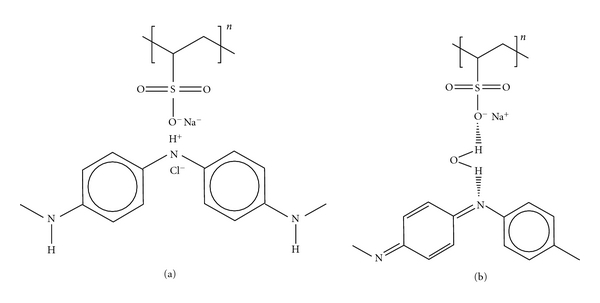
Representation of the mechanisms of the adsorption process proposed for the PANI and PVS: (a) adsorption in the presence of electric charge and (b) adsorption in the absence of electric charge [26].

 When PVS was replaced by cashew gum (Figure 3(b)) the fourth oxidation process disappeared because the interaction between PANI and PVS disappeared as well. The interaction between these PANI and PVS can occur by two distinct mechanisms, similarly as proposed by Raposo & Oliveira for LbL films of poly(*o*-methoxyaniline) (POMA) and PVS [26]. This process can occur through the establishment of links between PANI and PSV in the presence of electrical charge (Scheme 2(a)) and/or in the absence of electrical charge through the formation of networks of water molecules from the PANI present in solution with PVS (Scheme 2(b)).

Probably the adsorption processes proposed for the PANI and PVS must be somehow related to the oxidation process observed in the region of 0.86 V in Figure 3(a). In our studies we observed that the presence of cashew gum significantly decreases the degradation of the polymer in acid media, which is observed in films PANI-PA/PVS around 0.43 V (shown in Figures 2(a) and 3(a)).

**Figure 4:**
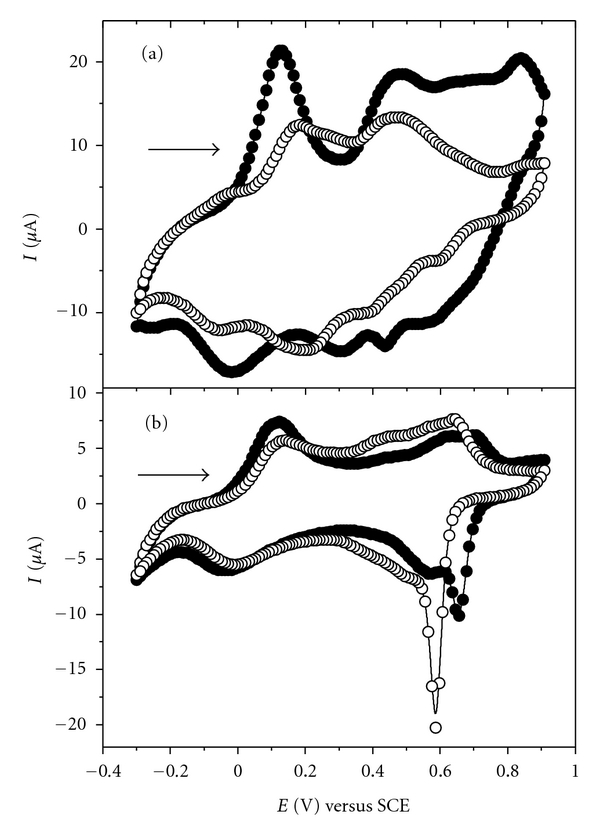
Cyclic voltammograms for LbL films from (a) (PANI-PA/PVS)_6_ and (b) (PANI-PA/CG)_6_ after five and twenty (5th cycle —*⬤*— and 20th cycle —◯—) successive cycles in 0.1 mol L^−1^ HCl solution, at 50 mV s^−1^.

In Figure 4 the cyclic voltammograms of the films with 6 bilayers of PANI-PA/PVS (Figure 4(a)) and PANI-PA/CG (Figure 4(b)) the 5th and the 20th successive cycles of scanning potential at 50 mV s^−1^ are shown. The electrode polarization until 0.90 V led to a more visible degradation of the conducting polymer in system PANI-PA/PVS. Cyclic voltammograms for these films show the presence of intermediate redox process around 0.43 V, which is related to soluble products (radical benzoquinone/hydroquinone) formed during the acid degradation of polyaniline [25] accompanied by a decrease of the current values in all the processes observed. On the other hand, the process for proton expulsion and anion uptake seems more stable for the PANI-PA/CG film even after twenty successive cycles.

Therefore, the polyaniline suffers acid degradation, promoted by the high polarization potential and by electrolyte of HCl, and in the case in the PANI-PA/PVS system this process is enhanced by the presence of PA modifier. On the other hand, when PVS was replaced by CG the degradation process of PANI was reduced indicating that the cashew gum protects the film from the degradations processes mentioned above, presenting a greater stability during scanning in an acid medium and polarizations at 0.90 V compared to PANI-PA/PVS or PANI/PVS films studied. Therefore, the cashew gum acts as a kind of antioxidant for polyaniline. Previous works from our group [8] showed that LbL films of POMA and gums have greater stability during scanning potential in an acid medium than films POMA/PVS and that the gums as chichá (*Sterculia striata*) and angico (*Anadenanthera macrocarpa *B.) act protecting the polymer film from this degradation.

The process of charge transfer from the ITO-modified electrode with PANI-PA/CG film containing 6 bilayers was studied by varying the scan rate (*v*) in the range of 10 to 150 m V s^−1^ (data not shown). In these conditions it was observed that the values of the anodic peak current (*I*_pa1_) increased linearly with scan rate for the film PANI-PA/CG, according to the equation *I*_pa1_(*μ*A) = −1.92(±0.76) + 0.30(±0.010) *v* (mV s^−1^), with a linear correlation, *r* = 0.998. This behavior indicates a redox process of electroactive species that are strongly adsorbed on the ITO surface, confirming that the electrochemical reaction is controlled by a kind of electron hopping mechanism of charge transfer on the electrode surface [33].

**Figure 5:**
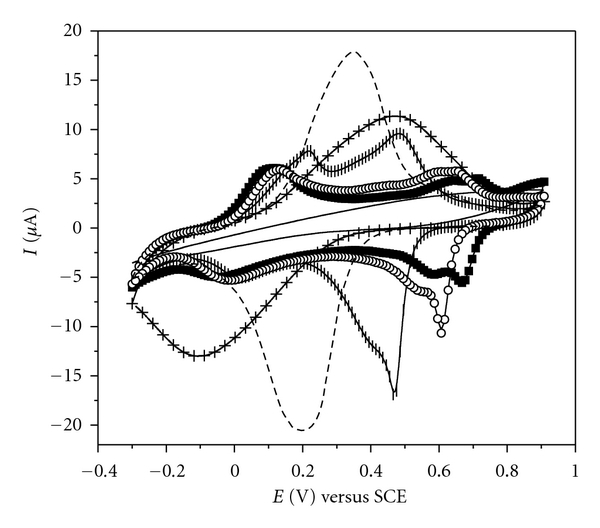
Cyclic voltammograms for LbL films from (PANI-PA/CG)_6_ in HCl electrolyte at different pHs (—■— pH 1 initial; —|— pH 2; - - - pH 3; —+— pH 4; — pH 5; —*⚪*— pH 1 final). Scan rate: 50 mV s^−1^.

The dependence on pH solution of the electrochemical behavior of PANI-PA/CG film was studied and shown in Figure 5. When increasing the pH from 1 to 2 an approximation of PANI redox process occurs, and in pH 3.0 and 4.0 it is possible to observe the overlap of two redox processes at 0.30 V and 0.20 V for pH 3.0 and at 0.48 V and −0.10 V for pH 4. On the other hand at pH 5 the PANI-PA/CG film no longer presents any electrochemical activity. However, when the same film was scanned again at pH 1.0 it presented the characteristic processes of electroactive polyaniline with potential and current values similar to the first scan done at pH 1.0 before variations in pH described above. This reversibility in the electrochemical behavior in function of the pH media is not observed in films of PANI/PVS or PANI-PA/PVS. Thus, the results suggest that in the case of PANI-PA/CG films, the cashew gum acts as a stabilizing element of the polyaniline.

LbL films of cashew gum containing modified polyaniline with PA dopant are very interesting for applications in electrochemical sensors due to their high reproducibility and stability. Thus, the self-assembled film of PANI-PA/CG containing 6 bilayers was selected to be applied to detect dopamine (DA), an important neurotransmitter in the central nervous system of mammals [34, 35].

### 3.2. Study of Modified Electrode with PANI-PA/CG Nanocomposite as Biosensor

**Figure 6:**
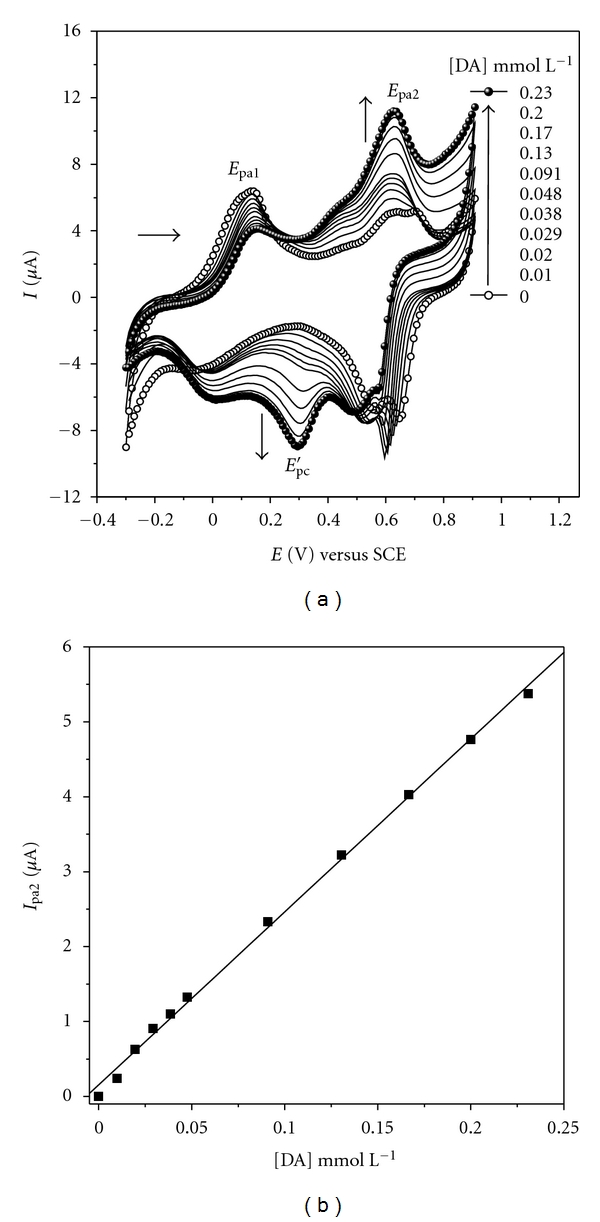
(a) Cyclic voltammograms for LbL films from (PANI-PA/CG)_6_ in 0.1 mol L^−1^ HCl solution in the presence of DA at concentrations ranging from 0.01 mmol L^−1^ to 0.23 mmol L^−1^ (from bottom to top). (b) The dependence of peak current (*I*_ap2_) on the DA concentration. Scan rate: 50 mV s^−1^.

Figure 6(a) shows the cyclic voltammograms for the LbL film with 6-bilayers of PANI-PA/CG in different concentrations of dopamine (DA). In this figure a redox couple (*E*_pa2_/*E*_pc_′) appears associated with oxidation of dopamine in dopamine quinone at 0.63 V and its reduction at 0.29 V [34, 35].

The anodic current peak (*I*_pa2_) increases linearly for concentrations of DA between 0.01 and 0.23 mmol L^−1^ for the modified electrode, as shown in the calibration curve in Figure 6(b). The calibration equation obtained by linear regression is *I*_pa2_(*μ*A) = 0.15(±0.043) + 23.10(±0.37) [DA]/mmol L^−1^ with a correlation coefficient (*r*) equal to 0.998 (for *n* = 10) and sensibility of 23.10 *μ*A mmol^−1^ L. The limit of detection (LD) of 1.5 × 10^−8^ mol L^−1^ was estimated using 3*σ*/slope ratio, where *σ* is the standard deviation calculated from 10 blank samples and slope refers to the slope of the calibration curve, according to the IUPAC recommendations [36]. The modified electrode presented reversibility after washing and a good repeatability for DA determinations with relative standard deviation, RSD = 6% in five determinations in the presence of 0.23 mmol L^−1^ DA.

Table 1 summarizes the analytical performance on different modified electrodes for DA detection by electrochemical process [27–32]. The lowest LD obtained was observed for film from (PANI-PA/CG)_6_, revealing itself as a competitive electrode for this analysis when compared to other modified electrodes.

The relationship of peak current (*I*_pa2_) for DA oxidation with the scan rate (*v*) has been investigated for 6 bilayers from PANI-PA/CG film in 0.1 mol L^−1^ HCl solution in the presence of 0.23 mmol L^−1^ DA (data not shown). Under these conditions a linear relationship between anodic current peak (*I*_pa2_) and the square root of scan rate (*v* ^1/2^) for the PANI-PA/CG film was found according to the equation *I*_pa2_(*μ*A) = −0.27(±0.14) + 2.32(±0.02) *v* ^1/2^ (mV s^−1^)^1/2^, *r* = 0.999. This behavior indicates that the electrocatalytic process of electron transfer is controlled by dopamine diffusion from the solution to the redox sites of PANI-PA/CG films [37].

**Figure 7:**
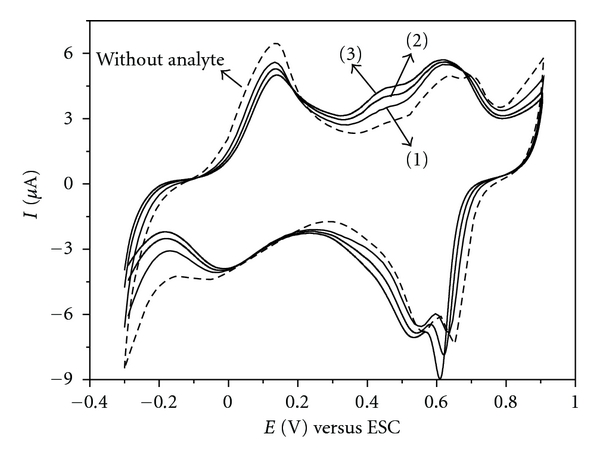
Cyclic voltammograms for LbL films from (PANI-AP/GC)_6_ film in the absence and presence of 0.01 mmol L^−1^ DA containing ascorbic acid at a concentration of (1) 0.01 mmol L^−1^ (2) 0.02 mmol L^−1^ and (3) 0.03 mmol L^−1^ in 0.1 mol L^−1^ HCl solution, at 50 mV s^−1^.

In order to investigate the selectivity of the PANI-PA/CG modified electrode, we tested the simultaneous detection of 0.01 mmol L^−1^ DA in different ascorbic acid (AA) concentrations, the natural interfering of DA [34]. It is noted in Figure 7 that when we increase the concentration of AA a proportional increase in the current values at 0.45 V is accompanied. Moreover, in the oxidation potential of dopamine, 0.63 V, the increase of current with the addition of AA is minimal, showing that oxidation of dopamine at the surface of modified electrode is slightly affected by trace amounts of AA (Figure 7).

Additionally it is important to observe that this new nanocomposite using cashew gum, which is a natural and biocompatible polymer, in the multilayer structure, gives rise to new applications as biosensor [38, 39]. Additionally, this film could be used in wound repair because it has been shown that very small exogenously applied electrical currents produce a beneficial therapeutic result for wounds [40]. In this work we propose a formation of electroactive nanocomposite, which can be a potential tool for wound repair when associated with the electrostimulation. 

## 4. Conclusions

The electrochemical profiles observed for the films studied showed the redox intrinsic characteristic transitions of polyaniline. The presence of CG increases the electrochemical stability of the film, suggesting that it acts by protecting the conductive polymer from acid degradation. LbL films of PANI/PVS and PANI-PA/PVS show an oxidation process around 0.86 V, which can be related to interaction between PANI and PVS. Detailed studies showed that the electrochemical reaction in the PANI-PA/CG film is governed by a charge transfer mechanism at the surface electrode via electron hopping. The ITO-modified electrode with PANI-PA/CG film showed high reproducibility and stability, encouraging its use as a sensor of DA. This modified electrode was able to detect electroactive molecule of DA around 0.63 V in detection limits consistent with the pharmaceuticals formulations.

## Figures and Tables

**Table 1:** Comparative performance of different electrodes for dopamine determination.

Electrode	Method	Dynamic range (mmol L^−1^)	LD (mol L^−1^)	Reference
ITO modified with LbL film from (PANI-PA/CG)_6_	CV^a^	0.01–0.23	1.5 × 10^−8^	This work
ITO modified with LbL film from (PAH/Chichá gum/PAH/NiTsPc^b^)_5_	CV	0.3–250	1.05 × 10^−5^	[27]
ITO modified with LbL film from ([PAMAM-MWCNTs]^c^/NiTsPc)_3_	CV	2.5 × 10^−3^–0.24	5.4 × 10^−7^	[28]
GCE^d^/MWCNTs modified with LbL film from (Nafion/[PVI-dmeOs]^e^)_3_	DPV^f^	1.0 × 10^−4^–0.01	5.0 × 10^−8^	[29]
GCE modified with poly(flavin adenine dinucleotide)	CV	2.5 × 10^−6^–4.0 × 10^−5^	5.0 × 10^−9^	[30]
ITO modified with LB film of (PANI/Rupy^g^)_21_	CV	0.04–1.2	4.0 × 10^−5^	[31]
GCE modified with (LiTCNE/PLL)^h^ membrane	DPV	1.0 × 10^−5^–0.01	5.0 × 10^−10^	[32]

^a^Cyclic voltammetry.

^b^Tetrasulfonated metallophthalocyanine of nickel.

^c^Polyamidoamine-multiwalled carbon nanotubes.

^d^Glassy carbon electrode.

^e^Poly(vinylimidazole)-Os(4,4′-dimethylbpy)_2_Cl.

^f^Differential pulse voltammetry.

^g^Ruthenium complex *mer*-[RuCl_3_(dppb)(py)] (dppb = PPh_2_(CH_2_)_4_PPh_2_; (py) = pyridine).

^h^Lithium tetracyanoethylenide/poly-L-lysine.
